# Circulating Tumor DNA Testing Opens New Perspectives in Melanoma Management

**DOI:** 10.3390/cancers12102914

**Published:** 2020-10-10

**Authors:** Alessandra Sacco, Laura Forgione, Marianeve Carotenuto, Antonella De Luca, Paolo A. Ascierto, Gerardo Botti, Nicola Normanno

**Affiliations:** 1 Cell Biology and Biotherapy Unit, Istituto Nazionale Tumori-IRCCS-Fondazione G. Pascale, 80131 Naples, Italy; a.sacco@istitutotumori.na.it (A.S.); l.forgione@istitutotumori.na.it (L.F.); m.carotenuto@istitutotumori.na.it (M.C.); a.deluca@istitutotumori.na.it (A.D.L.); 2 Department of Melanoma, Cancer Immunotherapy and Development Therapeutics, Istituto Nazionale Tumori IRCCS Fondazione Pascale, 80131 Napoli, Italy; p.ascierto@istitutotumori.na.it; 3 Scientific Direction, Istituto Nazionale Tumori IRCCS Fondazione Pascale, 80131 Napoli, Italy; g.botti@istitutotumori.na.it

**Keywords:** ctDNA, melanoma, liquid biopsy, prognosis, prediction, patient stratification

## Abstract

**Simple Summary:**

Melanoma, like other solid tumors, releases DNA molecules that are referred to as circulating tumor DNA (ctDNA), into the blood and other biological fluids. ctDNA analysis performed with molecular biology techniques can provide important information on the aggressiveness of the disease and its genetic characteristics. This review aims to highlight all the possible clinical applications of ctDNA analysis that can contribute to an improvement in the diagnosis and therapy of melanoma.

**Abstract:**

Malignant melanoma accounts for about 1% of all skin cancers, but it causes most of the skin cancer-related deaths. Circulating tumor DNA (ctDNA) testing is emerging as a relevant tool for the diagnosis and monitoring of cancer. The availability of highly sensitive techniques, including next generation sequencing (NGS)-based panels, has increased the fields of application of ctDNA testing. While ctDNA-based tests for the early detection of melanoma are not available yet, perioperative ctDNA analysis in patients with surgically resectable melanoma offers relevant prognostic information: i) the detection of ctDNA before surgery correlates with the extent and the aggressiveness of the disease; ii) ctDNA testing after surgery/adjuvant therapy identifies minimal residual disease; iii) testing ctDNA during the follow-up can detect a tumor recurrence, anticipating clinical/radiological progression. In patients with advanced melanoma, several studies have demonstrated that the analysis of ctDNA can better depict tumor heterogeneity and provides relevant prognostic information. In addition, ctDNA testing during treatment allows assessing the response to systemic therapy and identifying resistance mechanisms. Although validation in prospective clinical trials is needed for most of these approaches, ctDNA testing opens up new scenarios in the management of melanoma patients that could lead to improvements in the diagnosis and therapy of this disease.

## 1. Introduction

Melanoma is an aggressive and deadly disease that is responsible for the largest number of skin cancer-related deaths, although it comprises less than 10% of skin cancers. The high mortality rate of melanoma is due to the late diagnosis and to the highly metastatic potential of melanoma cells, which typically spread in different organs [1].

The therapeutic strategies for advanced melanoma have significantly improved in the past few years thanks to the introduction of targeted agents and immune checkpoint inhibitors. More recently, both targeted therapy and immunotherapy have shown to reduce the rate of recurrence in patients with resectable, locally advanced disease. However, mechanisms of intrinsic and acquired resistance greatly limit the activity of therapies in melanoma, especially in the most advanced phases of disease. For this reason, the identification of non-invasive biomarkers could be key for facilitating early detection, patient stratification, and monitoring the response and resistance to therapy.

The testing of circulating tumor DNA (ctDNA) is emerging as a relevant tool for the diagnosis and monitoring of cancer [2]. Almost every tumor type releases DNA that can be isolated from the peripheral blood or other body fluids. Increasing evidence suggests that ctDNA recapitulates the genomic complexity of the tumor, and, therefore, it might represent a non-invasive tool for assessing its genomic profile. In addition, the non-invasive or minimally invasive nature of ctDNA testing allows repeated measurements over time, thus ensuring the possibility to evaluate the response to therapy and to monitor the genomic evolution of the disease under the pressure of the therapies [3]. Importantly, in patients with advanced disease and multiple localizations, the analysis of ctDNA might allow a better profiling of the heterogeneity of the disease as compared to the testing of tumor tissue derived from single metastases.

**Figure 1:**
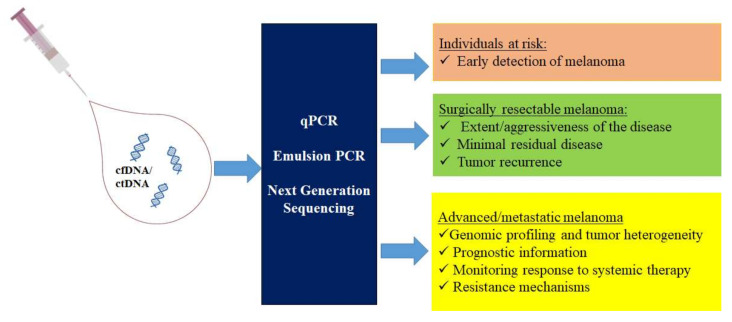
Applications of circulating tumor DNA (ctDNA) analysis in melanoma.

The availability of new methods for the genotyping of ctDNA and the development of next generation sequencing (NGS)-based panels with increased sensitivity have significantly amplified the applications of ctDNA testing in the management of cancer patients [4,5]. In this review, we will describe and discuss the current knowledge as well as the potential and future applications of ctDNA analysis in the different stages of melanoma, from early diagnosis to the genomic profiling of the tumor and the monitoring of the response to therapy (Figure 1).

## 2. Circulating Tumor DNA and Circulating Cell-Free DNA

Circulating cell-free DNA (cfDNA) is highly fragmented (~166 bp) double-stranded DNA that freely circulates in body fluids including the plasma, serum, urine and cerebrospinal fluid. The release of cfDNA from damaged or dead cells occurs in normal physiological conditions, with most of the cfDNA being shed by white blood cells [6]. cfDNA has a relatively short half-life, ranging from 16 to 139 min.

An increase in cfDNA levels is observed in physiological conditions, as well as in pathological conditions, including cardiovascular diseases, heart failure, infections and cancer [7]. In cancer patients, a proportion of cfDNA, defined as ctDNA, comes from primary tumors, metastatic sites and/or circulating tumor cells. The ctDNA is released into the systemic circulation as a result of tumor cell apoptosis and/or necrosis, but the exact mechanism by which tumor cells release DNA is not yet fully clarified [2]. In the blood of cancer patients, ctDNA is only a fraction of the cfDNA, which includes DNA released from non-tumor cells, cells of the tumor microenvironment, and other cell types including stromal cells, endothelial cells and lymphocytes [7].

**Figure 2:**
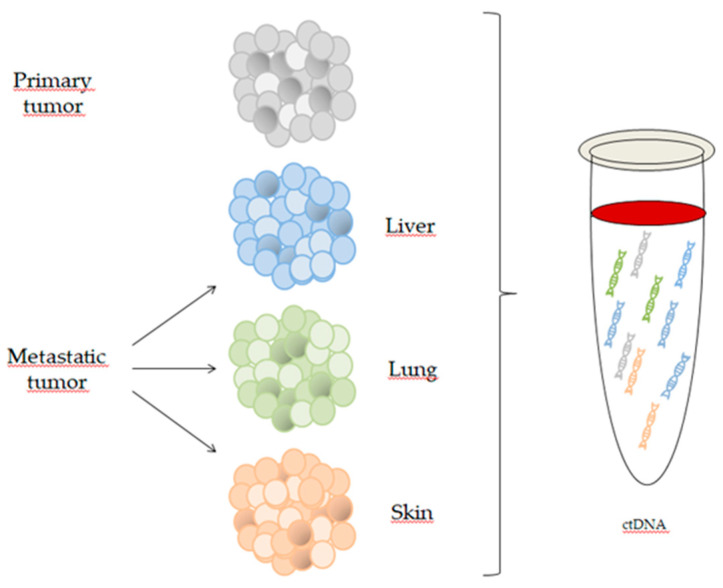
Circulating tumor DNA (ctDNA) reflects the genomic profile of different tumor localizations in metastatic melanoma and better recapitulates the heterogeneity of the disease.

In this regard, the ctDNA fraction of the total cfDNA isolated from the peripheral blood can vary from <1% to 90% and is generally correlated with the tumor burden, although the localization of the tumor and the histological type might affect ctDNA release in the bloodstream (Figure 2) [8]. For this reason, the analysis of ctDNA in a background of cfDNA might be difficult, particularly in early stage cancer.

cfDNA can be isolated from different body fluids. When cfDNA is obtained from the peripheral blood, plasma is preferred to serum, especially because the large amounts of wild-type DNA released by white cell lysis during clotting can cause a further dilution of ctDNA. To prevent clotting and DNase I activity, it is recommended to use ethylenediamine-tetraacetic acid (EDTA) tubes or tubes containing formaldehyde-free preservative reagents for blood collection [9]. When EDTA-containing tubes are used to collect blood, plasma separation must be performed within 4 h of drawing to prevent the lysis of leukocytes [10,11].

## 3. Methods for cfDNA Testing

Because of the possible dilution of tumor DNA in normal DNA, highly sensitive methods are needed to detect variants at low mutant allelic frequency (MAF) in the cfDNA. In this regard, sensitive methodologies have been developed to detect and quantify rare variants in the blood of cancer patients, with analytical sensitivity ranging between 0.005 and 5% [12]. These technologies can be classified into three groups: quantitative PCR (qPCR), emulsion-PCR and massively parallel sequencing, more commonly defined as NGS.

Melanoma most frequently harbors genomic alterations in *BRAF*, *NRAS*, *KIT* or *NF1* [13,14]. In particular, several efforts have been made to develop technologies capable of detecting *BRAF V600* variants in cfDNA, because of the availability in clinical research and clinical practice of drugs targeting tumors carrying these mutations.

A number of different approaches based on qPCR have been developed in order to improve its sensitivity for the analysis of rare variants. Allele-specific PCR (AS-PCR), allele-specific amplification refractory mutation system PCR (ARMS) and peptide nucleic acid (PNA)-based PCR have demonstrated, in different studies, analytical sensitivity up to 0.001% for the detection of the *BRAF-V600E* mutation [15].

Comparing the performance of some qPCR tests in detecting mutant *BRAF* in the plasma from stage IIIc–IV melanoma patients, Denis et al. observed that the Therascreen *BRAF* RGQ kit (Qiagen) was able to detect *BRAF* mutations in 73.7% of plasma samples from patients with BRAF-positive tumors. Similar results were obtained with the ct*BRAF* Mutation Detection Kit (Entrogen), which showed a positive percent agreement (PPA) of 68.4%. In all patients with wild-type *BRAF* tumors, the test was negative for cfDNA with both techniques [16].

In a recent study, the Cobas *BRAF/NRAS* (Roche) mutation test was used to analyze the ctDNA of 68 patients with stage III or IV melanoma. The test is an allele-specific, real-time PCR assay for the qualitative detection and identification of exon 11 and 15 mutations in the *BRAF* gene and exon 2, 3 and 4 variants of the *NRAS* gene from tumor tissue or plasma samples. The expected mutations were detected in the plasma of 34/68 patients (50% sensitivity), and the results obtained by the Cobas analysis were similar to those obtained by digital PCR (dPCR) [17]. Importantly, the sensitivity of the test significantly correlated with the stage. In fact, 64% of the tumor-positive stage IV patients were also positive at ctDNA analysis, while the sensitivity dropped to 19% for stage III melanoma patients. The specificity was 100%.

Recently, Biocartis (Belgium) developed a new fully automated platform, designated Idylla, for detecting the major *NRAS*, *BRAF*, *KRAS* or *EGFR* mutations in either ctDNA or genomic DNA isolated from tissue. The test allows the detection of *BRAF V600* mutations in plasma with up to three mutant copies per PCR reaction with an analytical sensitivity of 0.01% [18]. A 75% clinical sensitivity has been reported in stage IV melanoma patients [19].

Emulsion-PCR-based methodologies, such as dPCR, droplet digital PCR (ddPCR) and BEAming (which stands for beads, emulsion, amplification and magnetics) have a greater sensitivity as compared with qPCR, overcoming the problems due to the low levels of cfDNA and the low MAF of the variants [2,5]. In fact, BEAMing- and ddPCR-based assays can detect and enumerate mutant and wild-type DNA at ratios greater than 0.01% [15]. An additional advantage of the ddPCR technique is the lower sensitivity to clinically relevant inhibitors (SDS, EDTA and heparin) than qPCR [20,21]. A ddPCR *BRAF-V600E* test detected the variant in 84.3% of plasma-derived cfDNA obtained from advanced melanoma patients [22], while a sensitivity >75% for the detection *of BRAF-V600E/V600K* mutations in cfDNA was observed using a BEAMing-based assay [23].

Although qPCR and emulsion-PCR have a high sensitivity and specificity, both these methodologies have the limitation of being able to interrogate only a few loci for analysis. Therefore, the use of these techniques is limited to the screening of the most frequent known mutations. In addition, they cannot provide information on the genetic evolution of the tumor that might be relevant for therapeutic intervention, such as the identification of resistance mutations.

Many of these limitations are overcome by NGS, which is a high-throughput screening method that allows the simultaneous analysis of multiple genes and the detection of novel alterations, including low-frequency mutations. However, sensitivity represented, for a long time, a limiting factor for using NGS to test cfDNA. In this respect, targeted-sequencing panels are more suitable compared with whole-genome sequencing (WGS) or whole-exome sequencing (WES) due to their higher sensitivity, and reduced turnaround time and cost [5]. Novel NGS technologies have been recently developed for the specific analysis of cfDNA. These methods, by using molecular barcoding and improved bioinformatics pipelines [24,25,26,27], can detect variants at MAF < 1%. Tagged-Amplicon deep sequencing (TAm-seq), Safe-Sequencing System (Safe-SeqS), CAncer Personalized Profiling by deep sequencing (CAPP-Seq), and Ampliseq are examples of NGS technologies used for the targeted sequencing of cfDNA [4]. The limit of detection of these target panels depends on the quantity of cfDNA used for the preparation of libraries. To obtain high-quality libraries, about 20–30 ng of cfDNA is recommended, which can be commonly obtained from 4–5 mL of plasma.

Targeted-sequencing panels of different sizes, from a few mutations to hundreds of genes, are available both as a service and sold by different providers for independent laboratories [12]. Different labs developed custom NGS panels for cfDNA testing in melanoma patient. Although the specificity of NGS panels has significantly improved over time, the concordance between cfDNA and tissue testing ranges between 60% and 80% in most of the studies conducted in different tumor types. Several factors, including sequencing artifacts, tumor heterogeneity and clonal hematopoiesis, are involved in this relatively low concordance [28,29,30].

## 4. ctDNA for Early Detection of Melanoma

The chances of survival for melanoma patients significantly increase when the disease is diagnosed at an early clinical stage. Therefore, the improvement of melanoma early detection methods is fundamental for a proper management of this disease. However, several issues including the tumor heterogeneity, the tumor growth dynamics and the timing of metastasis, as well as the feasibility and cost of routinely applying liquid-biopsy techniques in clinical practice, make early detection challenging [31].

In a study aiming to define whether *BRAF V600E* could represent a suitable marker for melanoma detection, this mutation was identified only in ctDNA from stage III–IV melanoma patients, whereas, in early stages, it was undetectable in the majority of the cases [32]. While technical improvements might increase the sensitivity of mutation detection, the biology underlying ctDNA release and the relative low specificity of genetic alterations found in melanoma might still limit its potential use for early detection in an unselected population. For example, *BRAF-V600* mutations are present in only 50% of melanomas, while they can be found in a number of different tumor types [33]. Prospective studies might elucidate whether ctDNA analysis might be useful for monitoring melanocyte cell transformation in individuals at high risk.

NGS-based technologies might improve the use of cfDNA testing for the early detection of cancer. By testing a number of genetic alterations in a single analysis, these methods can significantly increase the sensitivity of the test. However, the detection of genetic alterations in the cfDNA might not have sufficient specificity for the early diagnosis of cancer. In this respect, the integration of information deriving from cfDNA analysis with other circulating biomarkers might significantly increase the sensitivity and specificity for the detection of melanoma in the early stages of development, as demonstrated for other cancer types [34]. In addition, incorporating such data into multiscale computational modeling platforms that can elaborate and integrate different types of information might allow individualized prediction for different cancer types.

## 5. Prognostic Value of ctDNA Testing in Patients with Surgically Resectable Melanoma 

A considerable fraction of patients with localized melanoma who undergo surgery with curative intent experience a relapse of the disease. Local recurrence and distant metastasis in melanoma patients are heavily dependent on tumor size. They occur in 50% of patients with a tumor thickness larger than 4 mm [35], in regional lymph nodes (50%), as local recurrence (20%), or at distant sites (30%) [36]. The recurrence of apparently localized disease following radical surgery and adjuvant therapy (if administered) is likely due to the persistence of minimal residual disease (MRD), a potential source of subsequent metastatic dissemination.

In this scenario, perioperative ctDNA testing in patients with surgically resectable melanoma can offer different, relevant prognostic information: (i) the detection of ctDNA before surgery might correlate with the extent and the aggressiveness of the disease; (ii) ctDNA testing after surgery and, eventually, adjuvant therapy might identify MRD; (iii) testing ctDNA during the follow-up after curative resection might detect a tumor recurrence at the molecular level, thus anticipating clinical or radiological recurrence. Indeed, given the possibility of minimally invasive repeated sampling, ctDNA testing can allow real-time monitoring during the course of the disease.

The detection and monitoring of MRD are widely established in patients with hematological malignancies [37]. More recently, several studies have shown that the perioperative detection of ctDNA in colorectal, breast and lung cancer patients has a strong negative prognostic significance [38,39,40].

Similar findings have been reported in patients with high-risk stage III melanoma [41]. In particular, pre-operative ctDNA levels were assessed by dPCR in 174 patients (119 in the discovery cohort and 55 in the validation cohort) with stage III melanoma who underwent complete lymph node dissection. Patients with a mutation in *BRAF*, *NRAS* or *cKIT* in tumor tissue were included in this study. ctDNA was detected in 34% of the patients in the discovery cohort and 33% in the validation cohort. The presence of ctDNA was significantly associated with tumor burden. More importantly, patients with detectable ctDNA had worse melanoma-specific survival in both the discovery cohort (Hazard Ratio, HR, 2.11) and the validation cohort (HR, 2.29), and this difference was confirmed via multivariate analysis (HR, 1.85).

The prognostic value of pre-operative ctDNA detection was confirmed in an additional study that enrolled stage III melanoma patients who were tested for ctDNA by dPCR [42]. However, this latter study demonstrated that in patients who did not receive adjuvant therapy, the post-operative detection of ctDNA was an even stronger predictor of shorter relapse-free survival (HR, 10). Interestingly, serial ctDNA testing in a cohort of patients with post-operative negative ctDNA could detect somatic mutations in plasma samples prior to clinical recurrence in 48% of the cases, with a 2 month median lead time. Similar findings have been recently reported in a small cohort of melanoma patients whose cfDNA was retrospectively analyzed [43].

The prognostic value of post-operative ctDNA testing was also explored in a retrospective analysis of plasma samples from 161 stage II/III patients carrying either a *BRAF* or *NRAS* mutation in their baseline-resected tumors. Patients with detectable ctDNA after surgery had a significantly increased risk of death compared to those with undetectable ctDNA, with HR = 2.50 for overall survival (OS) after adjustment for performance status [44]. The aforementioned studies are summarized in Table 1.

Although the above-summarized findings suggest a possible prognostic role of cfDNA testing in patients with resectable melanoma, these studies have several limitations. First, they included only patients with known mutations in a limited number of genes, thus excluding those patients that carry rare variants. In addition, while the specificity of the test is high, its sensitivity is relatively low because a significant fraction of ctDNA-negative patients have recurrences of the disease. In this respect, the use of NGS-based assays might increase the sensitivity of the test, being able to detect multiple mutations at the same time. Alternatively, the NGS testing of tumor tissue could be used to identify an adequate number of variants to test for in the cfDNA with highly sensitive dPCR-based assays.

## 6. cfDNA Testing as Tool to Support Treatment Decisions in Metastatic Melanoma Patients

The availability of different therapies makes highly relevant the use of biomarkers to stratify melanoma patients and identify the best therapeutic strategy for each individual patient. In this respect, cfDNA testing can provide relevant information at different levels: (i) the genomic profile of the disease and tumor heterogeneity, (ii) the prognosis, (iii) the response to therapy, and (iv) the development of resistance mechanisms.

### 6.1. cfDNA Testing for Genomic Profiling and Assessment of Tumor Heterogeneity

The analysis of cfDNA might represent an alternative to tumor tissue testing for the detection of predictive biomarkers. In this respect, a number of studies have demonstrated that qPCR and emulsion-PCR-based techniques can detect *BRAF* and *NRAS* mutations in plasma-derived cfDNA from patients with advanced melanoma (Table 2).

The sensitivity of the test ranged from 37.5% to 100%, although some of these studies enrolled a limited number of patients. By contrast, the specificity of the *BRAF/NRAS* testing of cfDNA was reported in a few studies and ranged between 75% and 100%. Taken together, these studies suggest that *BRAF* and *NRAS* mutations can be detected in the cfDNA from metastatic melanoma patients with a good specificity and an acceptable sensitivity.

The sensitivity of the cfDNA test might be affected by the localization of the tumor. In fact, patients with visceral, bone or lymph node involvement often display higher levels of ctDNA as compared with patients with extensive subcutaneous disease or brain metastases [53]. In agreement with these findings, the sensitivity of the *BRAF/NRAS* testing of cfDNA was found to correlate with the stage of the disease (higher in stage IV as compared with stage III) and the number and type of metastatic sites [17].

It has also been reported that the detectability of ctDNA is related to the nature of the mutated gene. Herbretau described a sensitivity of the cfDNA test of 36% for NRAS mutations and 66% for *BRAF* mutations [17]. In addition, mutations in the promoter region of the TERT gene were found at lower concentrations in the cfDNA as compared with other driver mutations, suggesting that they might be underrepresented in the cfDNA [54].

The sensitivity of cfDNA testing is also limited by the frequency of the mutant alleles in the context of wild-type DNA derived from normal cells. On the other hand, in selected cases, *BRAF* mutations have been identified through cfDNA analysis but not in the corresponding tumor tissue [49]. Such discordance is likely due to the heterogeneous expression of *BRAF* mutations in melanoma cells.

It has been demonstrated that driver mutations, including *BRAF* mutations in melanoma, are often clonal but can occasionally be subclonal [55]. This might lead to discordant results when different sites of the disease or different areas of the same tumor lesion are analyzed. In agreement with this hypothesis, discordant *BRAF* mutational statuses have been found between different sites of a primary tumor (intratumor heterogeneity), between a primary tumor and metastases, and between different metastases of the same patient [56,57]. In this scenario, the analysis of cfDNA is an approach that allows identifying mutations present in all the tumor sites of a given patient, thus better representing tumor heterogeneity [58]. Indeed, in cases with cfDNA positive for *BRAF* and BRAF mutation not detected in tumor tissue, the testing of additional samples from a different tumor area confirmed the presence of the *BRAF* variant [49].

Analysis of longitudinal samples from melanoma patients revealed that recurrent lesions might show a different *BRAF* mutational status over time as a consequence of tumor heterogeneity [59]. In the case of tumor relapse, the assessment of *BRAF* mutation status with a liquid biopsy might represent a non-invasive approach to confirming the mutational status of the disease.

Because the *BRAF-V600* mutations are the only approved biomarker in melanoma, the majority of data on genome profiling with liquid biopsies are based on the use of assays specific for these variants. However, NGS-based technologies might provide a better portrait of the genomic landscape and heterogeneity of melanoma, which might be relevant for therapeutic purposes. For example, it has been described that MITF and TP53 alterations are more frequent in patients with rapid progression of the disease following targeted therapy, while NF1 alterations are more common in cases with complete responses [60].

### 6.2. Prognostic Value of cfDNA Testing in Metastatic Melanoma

The identification of genetic alterations in cfDNA and the assessment of ctDNA levels before the administration of any systemic treatment may provide important prognostic information for patients with metastatic melanoma. In fact, a number of studies have demonstrated that high baseline levels of ctDNA correlate with a worse prognosis in metastatic melanoma patients treated with targeted therapy [22,23,45]. While the correlation between baseline ctDNA levels and survival is in line with previous findings in other tumor types including lung and colon carcinoma, in melanoma patients, a correlation of ctDNA levels with the response to treatment has been also reported. For example, in the phase II trial BREAK-2 enrolling *BRAF-V600E/K* metastatic melanoma patients to evaluate the clinical activity and the safety of the BRAF inhibitor dabrafenib, a correlation was found between the basal levels of *BRAF-V600E* in the cfDNA and tumor burden but not for the *V600K* variant, as assessed by using BEAMing technology [45]. Interestingly, patients with higher basal levels of cfDNA *BRAF-V600E* mutation showed a lower response rate and a shorter progression free survival (PFS) when treated with dabrafenib. In line with these findings, similar results were obtained in a larger cohort of 732 patients from four clinical studies of targeted therapy in metastatic melanoma (BREAK-2, BREAK-3, BREAK-MB and METRIC). Patients negative for *BRAF* mutations in the cfDNA had longer PFS and OS and higher rates of response to dabrafenib and trametinib, as compared with patients with detectable cfDNA *BRAF* mutations [23]. In multivariate analysis, the presence of *BRAF* mutations in the cfDNA was an independent predictive factor for shorter PFS in three out of four studies, and for shorter OS in one study.

Studies in smaller cohorts of patients further confirmed these findings. The quantification with ddPCR of mutant *BRAF-V600E* copies in cfDNA from 20 patients with metastatic melanoma showed a direct relationship between the *BRAF-V600E* copy numbers and clinical outcomes, where basal concentrations <216 copies/mL were significantly associated with better outcomes (OS = 27.7 months; PFS = 9 months) as compared with higher concentrations (OS = 8.6 months; PFS = 3 months) [22]. However, Schreuer did not find any correlation between the baseline levels of *BRAF* mutations in cfDNA and either survival or tumor responses in 25 patients receiving a combination of dabrafenib plus trametinib [61]. Importantly, this latter study enrolled patients who progressed on a previous line of therapy with a *BRAF* inhibitor, and the response rate was only 32%. The presence of a mechanism of resistance to a targeted therapy might represent a confounding factor for the prognostic role of ctDNA.

The basal levels of ctDNA are also a relevant prognostic marker in patients treated with immunotherapy. Seremet assessed ctDNA levels in 85 advanced melanoma patients using either the Idylla assay or a ddPCR test [18]. They found that patients in which the ctDNA was not detectable at baseline had longer PFS (HR, 0.47) and OS (HR, 0.37) as compared with patients with detectable ctDNA. Furthermore, high ctDNA levels (> 500 copies/mL) at baseline in the group of patients with progressive disease were indicative of a very poor clinical outcome [18]. However, an additional study exploring the prognostic role of ctDNA in melanoma patients receiving immune-checkpoint inhibitors suggested that the dynamics of the ctDNA were better associated with outcome than was the baseline ctDNA status [62].

Low baseline ctDNA levels were associated with higher response rates and longer PFS and OS in studies that included cohorts of patients treated with either targeted therapy or immunotherapy [17,46]. Importantly, the baseline levels of ctDNA before first-line therapy were confirmed via multivariate analysis to be an independent prognostic factor for OS, irrespective of treatment, in patients with stage IV or unresectable stage III metastatic melanoma [17]. Interestingly, ctDNA analysis in recurrent patients or during treatment (non-first line) was not associated with either PFS or OS. However, this latter analysis was limited to a very small cohort of patients.

The strong prognostic value of ctDNA levels in metastatic melanoma patients suggests that this parameter might better recapitulate the disease burden as compared with other prognostic factors. In this respect, several studies have demonstrated a correlation between ctDNA levels and the American Joint Committee on Cancer (AJCC) stage, number of metastatic sites, and serum levels of Lactate dehydrogenase (LDH) and S100 [49,63]. A significant correlation has also been reported between baseline ctDNA levels and metabolic tumor activity [43,53,63]. Interestingly, baseline ctDNA levels presented a better correlation with disease burden as measured by 2-[fluorine-18]-fluoro-2-deoxy-d-glucose positron emission tomography (FDG-PET) when compared with LDH [53]. However, subcutaneous and cerebral disease localization were associated with lower ctDNA levels, suggesting that in these cases, FDG-PET might better recapitulate tumor burden [53].

### 6.3. Monitoring Response to Therapy

The availability of predictive and prognostic biomarkers significantly improved the selection of appropriate therapeutic approaches in melanoma patients. Nevertheless, the response to both targeted therapy and immunotherapy is often heterogeneous, even in molecularly selected cohorts of patients. Therefore, the availability of tools to assess the response to therapy could further improve the therapeutic approaches for patients with metastatic melanoma. In this respect, the analysis of cfDNA could represent a non-invasive and repeatable technique for the early detection and monitoring of melanoma response and/or progression.

A number of studies have addressed the possibility of using the *BRAF* mutation testing of cfDNA to monitor the response to *BRAF* inhibitors [22] or combinations of *BRAF* and *MEK* inhibitors [51] in patients with *BRAF*-mutant melanoma. Although these studies enrolled a limited number of patients and different qPCR [19] or emulsion-PCR [22,51]-based techniques, they consistently found that response to therapy was associated with a significant decrease in the levels of *BRAF* mutations in cfDNA, while an increase in *BRAF* mutant DNA was observed at progression. Interestingly, the increase in ctDNA levels preceded the clinical progression of the disease in a significant fraction of patients, with a lead time up to 110 days [51,61].

The predictive value of monitoring the *BRAF* mutations in cfDNA using the Idylla test was also explored in a phase II trial of a combination of dabrafenib plus trametinib in patients with advanced *BRAF-V600*-mutant melanoma pre-treated with targeted therapy [61]. Upon analyzing plasma samples from 25 patients enrolled in this study, patients responding to therapy showed a significantly lower level of *BRAF-V600*-mutant ctDNA after 2 weeks of treatment as compared with non-responding patients. In addition, the persistent detection of *BRAF-V600*-mutant ctDNA after 2 weeks of therapy was correlated with a shorter PFS as compared with that for patients with undetectable ctDNA (1.8 months vs. 5.9 months; *p* = 0.001).

Some studies have reported a very early spike in ctDNA concentration occurring 24/48 h after starting treatment with *BRAF* inhibitors [19,64]. This early spike is probably related to a massive release of tumor DNA due to treatment-induced tumor cell lysis.

Taken together, these findings strongly suggest that monitoring *BRAF* mutations in the cfDNA could represent a valuable biomarker for assessing the early response to targeted therapy in melanoma patients.

Testing cfDNA could also provide important information for the evaluation of the response to immune-checkpoint inhibitors in melanoma and other cancers. In fact, the assessment of the response to immune therapies is sometimes difficult, due to the possible increase in the tumor lesions because of the immune reaction, which mimics a progression of the disease. Such pseudo-progression might lead to the suspension of an active treatment.

Few studies have addressed the potential of cfDNA testing for monitoring the response to immune-checkpoint inhibitors in patients with advanced melanoma. Two initial studies in small cohorts of patients receiving different immune-therapeutics (either PD-1 inhibitors or CTLA inhibitors or combinations) demonstrated a correlation between ctDNA dynamics and response to therapy [64,65]. In particular, levels of mutant DNA were found to decrease in the cfDNA of patients responding to treatment and to increase at or before tumor progression, using either NGS [65] or ddPCR [64].

The predictive value of ctDNA monitoring was next explored in a cohort of 76 patients with metastatic melanoma who received treatment with pembrolizumab or nivolumab monotherapy or in combination with ipilimumab [62]. Although ddPCR was employed in this study for the detection of the *BRAF*, *NRAS* and *cKIT* most frequent variants, the criterion used by the authors was only whether the ctDNA was detectable or not. Interestingly, the response rate in patients with detectable ctDNA at baseline but undetectable after 12 weeks of therapy was similar to that in patients with undetectable ctDNA at baseline and after 12 weeks (77% and 72%, respectively), and much higher as compared with that in patients with detectable ctDNA at baseline and after 12 weeks (6%). In addition, the first two groups had significantly longer PFS and OS as compared with the third group. The predictive value of ctDNA monitoring at 12 weeks was confirmed via multivariate analysis, thus suggesting that the clearance of ctDNA is a relevant predictive marker of response to immune-checkpoint inhibitors in metastatic melanoma. The same research group assessed the ability of cfDNA testing to identify pseudo-progression in a study including 125 metastatic melanoma patients who received anti-PD-1 antibodies alone or in combination with ipilimumab [66]. In this study, all nine patients with confirmed pseudo-progression had a favorable ctDNA profile, defined as ctDNA undetectable at baseline that remained undetectable, or detectable at baseline that became undetectable or decreased by at least 10-fold during treatment, thus introducing a quantitative criterion. By contrast, 18/20 patients with true progressive disease had an unfavorable ctDNA profile, with ctDNA detectable at baseline and during treatment. The dynamics of the ctDNA levels in the first month after treatment were also found to predict progressive disease or response to therapy in 21/24 stage III/IV melanoma patients receiving either immunotherapy or targeted therapy [63].

Finally, some studies in small cohorts of patients tried to establish quantitative thresholds to define patients with response or progression at ctDNA analysis [67,68]. Both these studies found a good correlation between an increase in ctDNA levels and progression of the disease, while ctDNA reduction was also observed in patients who experienced progressive disease. One of the limitations of these studies was a focus only on the few most frequent mutations in *BRAF*, *NRAS* and *cKIT*. By assessing a single mutation, the test might follow the dynamics of a single cell clone, which might not represent the behavior of the entire tumor in the case of tumor heterogeneity.

The above-summarized data suggest that cfDNA testing might represent an adequate tool for monitoring responses to immune-checkpoint inhibitors in melanoma. However, prospective clinical trials are required to validate this approach in the clinic and to demonstrate that the early detection of tumor progression might allow a better therapeutic strategy.

The use of NGS-based techniques might significantly increase the fraction of patients who can be monitored, improve the specificity and sensitivity of the test and provide information on tumor heterogeneity and the clonal evolution of the disease. For example, the NGS analysis of cfDNA from a patient with vaginal mucosa melanoma revealed the presence of two subclones with different responses to imatinib [69]. One subclone carried a KIT mutation and responded to imatinib, while the other had a KIT wild-type gene and did not respond to targeted therapy.

### 6.4. Identification of Mechanisms of Resistance

Combined targeted therapy with *BRAF* and *MEK* inhibitors is associated with a high response rate in melanoma patients who carry a *BRAF-V600* mutation [70]. However, most patients who initially respond will relapse during therapy due to mechanisms of acquired resistance. In many cases, acquired resistance to anti-*BRAF* therapy in melanoma patients is due to the reactivation of the *MAPK* pathway by genetic or epigenetic mechanisms [71]. However, other mechanisms of resistance have been described, including genetic alterations leading to the activation of the *PIK3CA* signaling pathway.

The feasibility of cfDNA testing in the assessment of acquired resistance to melanoma has been explored in a few studies. In a study that employed ddPCR to test *BRAF* and *NRAS* variants, *NRAS* mutations were detected in 3/7 melanoma patients progressing on targeted therapy with vemurafenib, dabrafenib or a dabrafenib/trametinib combination [46]. These data are in agreement with previous reports that described the frequent involvement of *NRAS* mutations, in particular, at codon 61 (*p.Q61K/R*), in acquired resistance to dabrafenib/trametinib combination therapy in *BRAF*-mutant metastatic melanoma patients [72]. Interestingly, *NRAS* mutations were detected in the cfDNA before the clinical and radiological progression of the disease.

By using the WES analysis of cfDNA, *NRAS* and *PIK3CA* mutations not present prior to therapy were identified in melanoma patients progressing on targeted therapy with *BRAF* and/or *MEK* inhibitors [73]. Multiple *NRAS* mutations were also found in the same cfDNA sample from a patient with progression, thus confirming the likely multi-clonal origin of acquired resistance to targeted therapies.

Acquired mutations in *NRAS* were also identified in metastatic melanoma patients at progression following dabrafenib-and-trametinib treatment using the targeted sequencing of cfDNA [53]. Interestingly, targeted-sequencing analysis identified two variants in *MAP2K1* and *PTEN* in a patient at progression, suggesting that these two genetic alterations might both contribute to resistance.

In addition to point mutations, other genomic alterations including *BRAF* gene amplification have been described as driving acquired resistance to *BRAF/MEK* inhibitors [74]. Indeed, the whole-exome sequencing and low-coverage whole-genome sequencing of cfDNA identified *BRAF* gene amplification in 2/3 patients at progression [53].

Evidence suggests that acquired resistance to targeted therapy is often polyclonal. In agreement with this hypothesis, distinct molecular alterations have been detected concurrently in the same tumor sample or among multiple tumor sites from the same melanoma patient progressing on targeted therapy [75]. Therefore, resistance to targeted therapy is associated with an increase in tumor heterogeneity and branched evolution that might be better depicted by using cfDNA testing as compared with tissue analysis. However, the ability of plasma-derived cfDNA testing to represent spatial heterogeneity was found to be limited in the presence of subcutaneous or brain metastases that shed limited amounts of ctDNA into the blood flow [53].

## 7. Conclusions and Future Perspectives

The data summarized in this article clearly show that cfDNA analysis can offer important information on the prognosis of patients with melanoma in different stages of the disease. Furthermore, cfDNA analysis could allow a more accurate identification of patient candidates for targeted therapy or immunotherapy, through the representation of tumor heterogeneity and the early identification of patients who do not respond to therapies. However, the possibility of transferring this information into daily clinical practice depends on the resolution of a series of technological, biological and clinical issues.

The introduction of NGS technologies for cfDNA analysis has increased the possibility of extending the test to a larger percentage of patients and has increased its clinical and analytical sensitivity. However, several NGS panels for cfDNA analysis are commercially available. For many of these panels, validation studies on adequate cohorts of biological samples are lacking, and external quality control programs are not yet available for these specific tests. Therefore, their introduction into clinical practice must be carried out with extreme caution, in order to avoid problems of false positives or negatives.

The use of NGS panels increases the sensitivity of the cfDNA test but also increases the possibility of identifying mutations associated with clonal hematopoiesis [29]. This possibility considerably limits the use of NGS techniques for applications such as early diagnosis or even monitoring of the disease. Although *BRAF* mutations have not been associated with clonal hematopoiesis to date, the identification of other genetic alterations could still pose problems in the clinical interpretation of the data. At present, the contemporary analysis of DNA derived from leukocytes appears as the only approach to overcoming this limit.

The analysis of cfDNA can provide information only on genetic alterations, which, alone, are not sufficient to represent the biological variability of neoplasms. In the era of precision medicine, the integration of the genomic profile with other biological omics, including transcriptomics, proteomics, metabolomics and epigenomics, that can be determined in biological fluids, represents a fundamental element for the advancement of knowledge on the pathogenesis and progression of cancer [76]. A biological multiomic pattern combined with clinical and radiological information, including radiomics, will allow the development of novel strategies for diagnostic, prognostic and therapeutic purposes [77]. In particular, the possibility of integrating different information could then be important for the early diagnosis of cancer, given the low specificity of genetic alterations for this specific application. In addition, the addition of multiple layers of clinical and biological information will allow a better stratification of patients, thus improving the clinical implementation of precision medicine. It must be emphasized that the integration of multiple omics information for patients’ stratification will require the development of appropriate disease modeling systems based on the use of machine learning [78].

The various potential applications of liquid biopsies in the early diagnosis of recurrence and in monitoring the response to therapy must be validated in prospective clinical trials. In fact, it will be necessary to demonstrate that the early detection of disease recurrence allows the development of therapeutic strategies that result in a decrease in the mortality of patients with early stage melanoma. At the same time, it must be demonstrated that the modification of therapy in patients with metastatic melanoma who do not respond in terms of reduced ctDNA results in better survival.

However, although there are many issues to be solved for the clinical implementation of cfDNA analysis, it can certainly be said that this technology opens up new scenarios in the management of patients with melanoma that could lead to important improvements in the diagnosis and therapy of this disease. In particular, the possibility of determining the overall profile of the genetic alterations of the neoplasm and of being able to evaluate its progression over time represents an important opportunity to improve the stratification of patients with melanoma for the purposes of therapeutic planning.

## Figures and Tables

**Table 1:** Main studies that addressed the prognostic value of ctDNA testing in patients with early (surgically resectable) melanoma.

Target Genes	AJCC Stage	Pre-/Post-Operative ctDNA Testing	N. of Patients	Clinical Outcomes	Reference
*BRAF, NRAS, cKIT*	III	Pre-operative	174	Presence of ctDNA associated with tumor burden and worse melanoma-specific survival.	Lee et al. [41]
*BRAF, NRAS, TERT*	III	Pre-operative	99	- Pre- and post-operative ctDNA detection correlates with shorter RFS and DMFS; - Post-operative ctDNA is an independent predictor of RFS and DMFS.	Tan et al. [42]
		Post-operative	68		
*BRAF, NRAS, TERT*	0–III	Post-operative	30	ctDNA detected at or before disease recurrence.	McEvoy et al. [43]
*BRAF, NRAS*	II/III	Post-operative	161	Presence of ctDNA predicts shorter DFI, DMFI and OS.	Lee et al. [44]

AJCC: American Joint Committee on Cancer; RFS: relapse-free survival; DMFS: distant metastasis-free survival; DFI: disease-free interval; DMFI: distant metastasis-free interval; OS: overall survival; ctDNA: circulating tumor DNA.

**Table 2:** Diagnostic performance of detecting *BRAF* and *NRAS* mutations in plasma using quantitative real-time PCR and emulsion-PCR-based techniques.

Reference	N° of patients	AJCC stage	Tissue mutation	Method	Specificity (%)	Sensitivity (%)
Ascierto et al. [45]	91	IV	*BRAF V600E* (*n* = 72)	Digital PCR (BEAMing Inostics)	-	79.2
			*BRAF V600K* (*n* = 19)		-	89.5
Sanmamed et al. [22]	20	IIIc–IV	*BRAF V600* (*n* = 20)	Droplet digital PCR (Biorad)	-	84.3
Gray et al. [46]	48	IV	*BRAF V600E* (*n* = 34) Healthy patients (*n* = 22)	Droplet digital PCR (Biorad)	100	64.7
			*BRAF V600K* (*n* = 8) Healthy patients (*n* = 23)		100	87.5
			*BRAF V600R* (*n* = 2) Healthy patients (*n* = 10)		100	100
			*NRAS Q61K* (*n* = 1) Healthy patients (*n* = 19)		100	100
			*NRAS Q61R* (*n* = 2) Healthy patients (*n* = 13)		84.6	100
			*NRAS Q61L* (*n* = 1) Healthy patients (*n* = 12)		75	100
Gonzalo-Cao et al. [47]	22	IV	*BRAF V600E* (*n* = 22)	qPCR LNA PNA clamp (in-house)	-	57.7
Santiago-Walker et al. [23]	661	IV	*BRAF V600E* (*n* = 661)	Digital PCR (BEAMing Inostics)	97.6	76.2
Chang et al. [48]	43	IIIc–IV	*BRAF V600E* (*n* = 20)	Droplet digital PCR (Biorad)	-	80
			*BRAF V600K* (*n* = 2)			
			*NRAS Q61K* (*n* = 4)			
			*NRAS Q61R* (*n* = 3)			
			*NRAS Q61L* (*n* = 2)			
Schreuer et al. [19]	16	IV	*BRAF V600* (*n* = 16)	Idylla (Biocartis)	100	75
Knol et al. [49]	29	IIIc–IV	*BRAF V600E* (*n* = 29)	Therascreen *BRAF* RGQ kit (Qiagen)	-	75.9
Denis et al. [16]	54	IIIc–IV	*BRAF V600* (*n* = 38) *BRAF* WT (*n* = 16)	Therascreen *BRAF* RGQ kit (Qiagen)	100	73.7
				ct*BRAF* Mutation Detection Kit (Entrogen)		68.4
				QuantStudio 3D system (Life Technologies)		58.8
Tang et al. [50]	58	I–II–III–IV	*BRAF V600E* (*n* = 58)	QuantStudio 3D system (Life Technologies)	-	74.1
Haselmann et al. [51]	187	III–IV	*BRAF V600* (*n* = 62) *BRAF* WT (*n* = 125)	Digital PCR (BEAMing Inostics)	91.2	90.3
Long-Mira et al. [52]	19	IV	*BRAF V600* (*n* = 10)	Idylla (Biocartis)	89	80
			*NRAS Q61/G12/G13* (*n* = 5)		100	79
			Double WT (*n* = 4)			
Seremet et al. [18]	85	III–IV1a, IV1b, IV–M1c	*BRAF V600* (*n* = 68)	Idylla (Biocartis)	-	47
			*NRAS Q61/G12/G13* (*n* = 22)		-	37.5
Herbreteau et al. [17]	48	III–IV	*BRAF V600* (*n* = 32)	Cobas *BRAF/NRAS* Mutation Test LSR kit (Roche)	-	50
			*NRAS* (*n* = 36)		-	

AJCC: American Joint Committee on Cancer.
